# Induction of Biomolecules in Mature Leaves of Terminalia arjuna Due to Feeding of Antheraea mylitta Drury

**DOI:** 10.1100/tsw.2004.143

**Published:** 2004-10-22

**Authors:** G. Abraham, G. Thomas, C.R. Babu

**Affiliations:** ^1^ Department of Botany, Allahabad Agriculture Institute-Deemed University, Allahabad, India; ^2^ Department of Biotechnology, Allahabad Agriculture Institute-Deemed University, Allahabad, India; ^3^ Department of Botany, University of Delhi, Delhi, India

**Keywords:** Terminalia arjuna, Antheraea mylitta, tasar silk, mature leaves, biomolecules, tannin, lipid peroxidation, trypsin inhibition

## Abstract

Terminalia *arjuna* is an important food plant of the tasar silkworm, *Antheraea mylitta* Drury. In this study, we investigated the induction of biomolecules in mature leaves of these plants subjected to insect feeding. Increase in total tannin content, lipid peroxidation, and trypsin inhibitor activity have been observed in mature leaves damaged by the insects. The growth rate of Vth instar larvae of *A. mylitta* fed on previously damaged foliage reduced by 87.1%. Induction of biomolecules for defense mechanisms in relation to herbivore damage has been discussed.

## INTRODUCTION

Tasar silk is superior in quality to other silks including “mulberry”, “muga”, and “eri” silks. It has a great international demand, but the productivity is too low and the demand exceeds production. *Terminalia arjuna*, commonly known as “arjun”, is an important food plant of the tasar silkworm, *Antheraea mylitta* Drury. Both plant and insect are found in the wild state and exploited by the tribes of the Jharkhand and Madhya Pradesh States of India for commercial tasar silk production. The tasar silkworm has two crop seasons. The first crop, or the seed crop, starts in June and extends until August. The second crop starts in September and lasts until November and is known as the commercial crop from which tasar silk is produced.

The interaction between insect and host plant is therefore an important factor that leads to the production of tasar silk. Insects play both beneficial and detrimental roles when they interact with plants. Phytophagy is a typical example where insects play a detrimental role by damaging the foliage. Plants, however, have evolved mechanisms to counter insect attack. Insect feeding leads to induced resistance in many plants[1,2]. Induced resistance, in turn, leads to enhanced synthesis of chemicals and secondary metabolites such as phenolics, alkaloids, and defensive proteins such as proteinase inhibitors[3,4,5,6,7]. It has been suggested that dietary composition of the ingested plant material such as protein and presence of proteinase inhibitors influence the profiles of digestive enzymes found in the midgut of phytophagous lepidopteran insects[8]. Thus, the present work was undertaken to elucidate the biochemical changes in the mature leaves of *T. arjuna* in response to herbivory by *A. mylitta*.

## MATERIALS AND METHODS

*T. arjuna* (DC) Wight & Arn (Combretaceae) used in the present study was collected from the Central Tasar Silk and Training Institute, Jharkhand, India. *T. arjuna* plants are primary food plants of the tasar silkworm, *A. mylitta,* and fully matured leaves were used in the study. Each plot consisted of approximately 30 trees and the leaves were collected randomly from at least 12 plots and were mixed. The leaves were divided into two categories, such as undamaged and damaged leaves, on the basis of insect damage. Undamaged leaves collected from plants not subjected to insect feeding served as control. Leaves subjected to 50% insect damage or more were designated as damaged leaves. Total tannin content of the leaves was determined and expressed as mg/tannin/g fresh weight[9]. Protein samples were prepared[10]. Trypsin inhibitor activity was assayed[11] and percentage of trypsin was calculated[12]. A thiobarbituric acid assay was performed to estimate the lipid peroxidation of foliar protein[13]. The amount of malonaldehyde present was calculated[14].

Insect growth rate was estimated by analyzing the weight gain of larvae fed on damaged and undamaged mature leaves. Plants were selected randomly on the basis of insect damage; 25 plants with damaged leaves eaten by IVth instar larvae of *A. mylitta* were selected and another 25 plants that were not subjected to insect feeding served as control. Damaged and undamaged mature leaves were excised from *T. arjuna* plants and were brought to the laboratory. Leaves were cleansed and placed in a plastic container (9 cm high with a few holes of 2-mm diameter in the plastic cover) with three layers of filter paper (Whatman No.1). One layer of filter paper was added after 24 h and another after 48 h. A newly molted Vth instar larva was placed in each container containing damaged leaves. Twelve such containers were employed in the study. Containers with undamaged leaves served as control. The containers were incubated at $32 \pm 1^{o}$C. Each larva was weighed at the beginning of experiment and again at 72 h of incubation.

## RESULTS AND DISCUSSION

Insect feeding markedly increased the total tannin content of the mature leaves (Table 1). A nearly twofold increase in total tannin content of damaged mature leaves of *T. arjuna* was observed as compared with undamaged leaves. An increase in the condensed tannin content in oak leaves from spring to autumn in response to insect feeding has been reported[15]. Tannins adversely affect the feeding, growth, and survival of certain insects[16,17]. Plant phenolics have negative effects on insects and they play important role in plant herbivore interactions[18]. The tannins may lead to avoidance of herbivores by inhibiting digestion due to complexing with plant proteins[19,20]. Generally, insect feeding leads to an increase in the concentration of defensive compounds having antiherbivore effects which leads to reduced acceptability by herbivores[21,22].

A comparatively high level of lipid peroxidation products was observed in damaged foliage (2.5-fold increase) as compared with foliage that has not been subjected to insect feeding (Table 1). Products of lipid peroxidation may deteriorate the leaf feed quality. Spider mite resistance in soya bean was correlated with enhanced levels of lipid peroxidation[23]. Decline in the quality of foliar protein due to lipid peroxidation has been observed in soya bean plants subjected insect feeding[24]. The products of lipid peroxidation may be toxic and function in resistance based on antibiosis[25,26].

**TABLE 1**
**A Profile of Tannin Content, Lipid Peroxidation, and Insect Growth Rate Due to Herbivory on the Mature Leaves of *T. arjuna***

| Type of Leaves | Tannin Content (mg/g fresh weight) | Lipid Peroxidation, Malonaldehyde (µmol/mg protein) | Insect Growth Rate (g) |
|---|---|---|---|
| Undamaged | 62.5 ± 2.14 | 0.92 ± 0.05 | 4.63 ± 0.219 |
| Damaged | 107.5 ± 4.6 | 2.23 ± 0.104 | 2.6 ± 0.115 |

Trypsins are a subfamily of hydrolytic endopeptidases known as serine proteinases that are used by lepidopteran insect pests for digestion of ingested protein[27]. Protein extracts of damaged mature leaves of *T. arjuna* showed inhibition of trypsin activity in the range of 80–90% (Fig. 1). High levels of foliar proteinase inhibitors were reported in soybean leaves due to Mexican bean beetle feeding[28]. Expression of proteinase inhibitors as defense to insect damage has been reported in many plants[29,30,31]. Induction of trypsin inhibitor may lead to changes in foliar protein and thereby cause a decline in the quality of foliar protein.

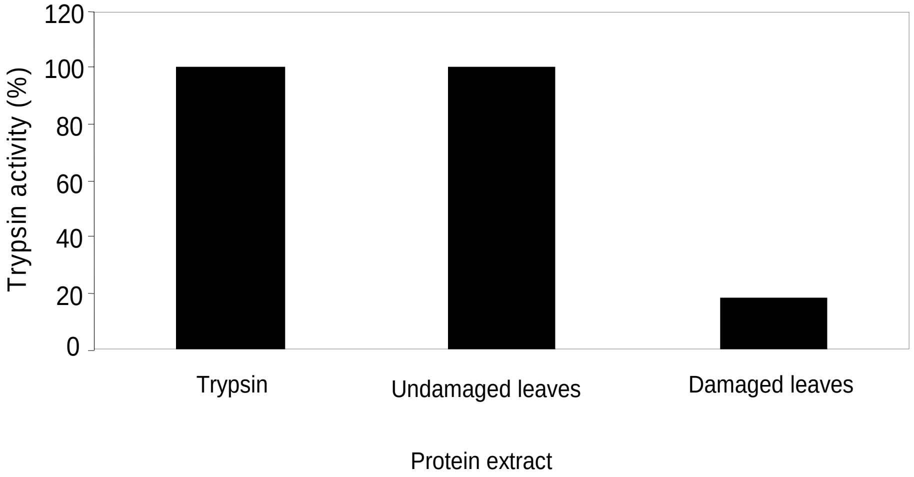

**FIGURE 1**. Trypsin inhibitor activity in mature leaves of *T. arjuna* in response to herbivory.

The growth pattern of insects that were fed on undamaged and previously damaged leaves showed significant differences (Table 1). A reduction in growth rate and development of soya bean loopers have been observed due to feeding on foliage subjected to prior damage by insects[32]. Damage-induced decline of leaf feed quality is possible due to increase in tannin levels, lipid peroxidation, and trypsin inhibitors. Therefore, in the present study, the accumulation of defensive biomolecules in damaged leaves of *T. arjuna* due to insect feeding might account for reduction in growth rate of Vth instar insects.

Based on the present investigation, it is concluded that feeding by *A. mylitta* leads to biochemical changes in mature leaves of *T. arjuna* and the complex biochemical mechanisms involved are increased levels of (a) tannins, (b) lipid peroxidation, and (c) trypsin inhibition due to induction of proteinase inhibitors. These factors might lead to less acceptability of foliage as evident from reduced growth rate of the insects. An increased production of biochemicals in response to herbivory has been well documented[33,34], however, virtually no information is available in the case of *T. arjuna*, which is an important food plant of the tasar silkworm. Induction of chemicals and related biochemical changes in the foliage may lead to deterioration in the nutritional quality of foliage. The biochemical characteristics of plants and selection of diet by herbivores are interlinked. The quality and quantity of silk produced by the silkworm depends largely on the nutritional status and biochemical constituents of the food plants[35]. Therefore, the quality of ingested food is an important factor in determining the quality of silk produced. Moreover, provisions of nutritionally high-quality food will keep larvae healthy, uniform in size, and able to produce good cocoons[36].

## ACKNOWLEDGMENT

Financial support from the Department of Biotechnology, New Delhi is gratefully acknowledged. We thank the Director and Staff of Central Tasar Research and Training Institute, Ranchi, Jharkhand for technical help.
